# An H3K4me3 reader, BAP18 as an adaptor of COMPASS-like core subunits co-activates ERα action and associates with the sensitivity of antiestrogen in breast cancer

**DOI:** 10.1093/nar/gkaa787

**Published:** 2020-09-28

**Authors:** Ge Sun, Chunyu Wang, Shengli Wang, Hongmiao Sun, Kai Zeng, Renlong Zou, Lin Lin, Wei Liu, Ning Sun, Huijuan Song, Wensu Liu, Tingting Zhou, Feng Jin, Zhongyan Shan, Yue Zhao

**Affiliations:** Department of Cell Biology, Key Laboratory of Cell Biology, Ministry of Public Health, and Key Laboratory of Medical Cell Biology, Ministry of Education, School of Life Sciences, China Medical University, Shenyang City 110122, Liaoning Province, China; Department of Cell Biology, Key Laboratory of Cell Biology, Ministry of Public Health, and Key Laboratory of Medical Cell Biology, Ministry of Education, School of Life Sciences, China Medical University, Shenyang City 110122, Liaoning Province, China; Department of Cell Biology, Key Laboratory of Cell Biology, Ministry of Public Health, and Key Laboratory of Medical Cell Biology, Ministry of Education, School of Life Sciences, China Medical University, Shenyang City 110122, Liaoning Province, China; Department of Cell Biology, Key Laboratory of Cell Biology, Ministry of Public Health, and Key Laboratory of Medical Cell Biology, Ministry of Education, School of Life Sciences, China Medical University, Shenyang City 110122, Liaoning Province, China; Department of Cell Biology, Key Laboratory of Cell Biology, Ministry of Public Health, and Key Laboratory of Medical Cell Biology, Ministry of Education, School of Life Sciences, China Medical University, Shenyang City 110122, Liaoning Province, China; Department of Cell Biology, Key Laboratory of Cell Biology, Ministry of Public Health, and Key Laboratory of Medical Cell Biology, Ministry of Education, School of Life Sciences, China Medical University, Shenyang City 110122, Liaoning Province, China; Department of Cell Biology, Key Laboratory of Cell Biology, Ministry of Public Health, and Key Laboratory of Medical Cell Biology, Ministry of Education, School of Life Sciences, China Medical University, Shenyang City 110122, Liaoning Province, China; Department of Cell Biology, Key Laboratory of Cell Biology, Ministry of Public Health, and Key Laboratory of Medical Cell Biology, Ministry of Education, School of Life Sciences, China Medical University, Shenyang City 110122, Liaoning Province, China; Department of Cell Biology, Key Laboratory of Cell Biology, Ministry of Public Health, and Key Laboratory of Medical Cell Biology, Ministry of Education, School of Life Sciences, China Medical University, Shenyang City 110122, Liaoning Province, China; Department of Cell Biology, Key Laboratory of Cell Biology, Ministry of Public Health, and Key Laboratory of Medical Cell Biology, Ministry of Education, School of Life Sciences, China Medical University, Shenyang City 110122, Liaoning Province, China; Department of Cell Biology, Key Laboratory of Cell Biology, Ministry of Public Health, and Key Laboratory of Medical Cell Biology, Ministry of Education, School of Life Sciences, China Medical University, Shenyang City 110122, Liaoning Province, China; Department of Cell Biology, Key Laboratory of Cell Biology, Ministry of Public Health, and Key Laboratory of Medical Cell Biology, Ministry of Education, School of Life Sciences, China Medical University, Shenyang City 110122, Liaoning Province, China; Department of Breast Surgery, the First Affiliated Hospital of China Medical University, Shenyang City 110001, Liaoning Province, China; Department of Endocrinology and Metabolism, Institute of Endocrinology, The First Affiliated Hospital of China Medical University, ShenyangCity110001, Liaoning Province, China; Department of Cell Biology, Key Laboratory of Cell Biology, Ministry of Public Health, and Key Laboratory of Medical Cell Biology, Ministry of Education, School of Life Sciences, China Medical University, Shenyang City 110122, Liaoning Province, China; Department of Endocrinology and Metabolism, Institute of Endocrinology, The First Affiliated Hospital of China Medical University, ShenyangCity110001, Liaoning Province, China

## Abstract

Estrogen receptor alpha (ERα) signaling pathway is essential for ERα-positive breast cancer progression and endocrine therapy resistance. Bromodomain PHD Finger Transcription Factor (BPTF) associated protein of 18kDa (BAP18) has been recognized as a crucial H3K4me3 reader. However, the whole genomic occupation of BAP18 and its biological function in breast cancer is still elusive. Here, we found that higher expression of BAP18 in ERα-positive breast cancer is positively correlated with poor prognosis. ChIP-seq analysis further demonstrated that the half estrogen response elements (EREs) and the CCCTC binding factor (CTCF) binding sites are the significant enrichment sites found in estrogen-induced BAP18 binding sites. Also, we provide the evidence to demonstrate that BAP18 as a novel co-activator of ERα is required for the recruitment of COMPASS-like core subunits to the *cis*-regulatory element of ERα target genes in breast cancer cells. BAP18 is recruited to the promoter regions of estrogen-induced genes, accompanied with the enrichment of the lysine 4-trimethylated histone H3 tail (H3K4me3) in the presence of E2. Furthermore, BAP18 promotes cell growth and associates the sensitivity of antiestrogen in ERα-positive breast cancer. Our data suggest that BAP18 facilitates the association between ERα and COMPASS-like core subunits, which might be an essential epigenetic therapeutic target for breast cancer.

## INTRODUCTION

Estrogen receptor alpha (ERα)/estrogen (17-β-estradiol, E2) signaling pathway exerts its various biological functions on normal mammary gland development, breast tumorigenesis, and tumor growth process (1–4). Although anti-estrogen has been widely adopted in ERα-positive breast cancer prevention and treatment (5,6), endocrine therapy resistance finally occurs due to many mechanisms, including depletion of ERα expression, activation of truncated ER isoforms, post-translational modification of ERα, abnormal regulation of ERα coactivators, and increased tyrosine kinase signaling (7–10).

Belonging to a nuclear receptor superfamily, ERα experiences conformational changes and trans-locates from the cytosol to the nucleus for binding to specific estrogen response elements (EREs) to regulate gene transcription upon estrogen treatment. Estrogen-dependent gene expression recruits a series of highly organized complexes, including various transcription factors (TFs), epigenetic enzymes, and chromatin remodelers to be essential for the breast cancer process (11–13). Estrogen-induced genes, such as *MYC*, *MTA3*, *CARM1* and so on, play vital roles in cell-cycle regulatory, tumor metastasis and endocrine treatment resistance (14–17). Thus, further exploring the molecular mechanism of regulation of the ERα-E2 signaling pathway in breast cancer would be extremely essential for the identification of the novel therapeutic targets of ERα-positive breast cancer and endocrine resistance.

Global genomic studies about histone modification indicate that H3K4 tri-methylation (H3K4me3) usually occurs nearby the transcription start site (TSS) of active genes (18–20). Histone methyltransferase (HMT) complexes, such as MLL1 to MLL4, have been recognized to be involved in histone H3K4 methylation. Some core proteins including ASH2L, WDR5, and DPY30 was named as a COMPASS-like complex (a complex of proteins associated with Set1, COMPASS), which interplays with modified histones or sequence-specific transcription factors to be recruited to chromatin (13,21,22). Concomitantly, the core subcomplex binds to specific histone methylated tails near the TSS region, subsequently facilitating the activate transcription (23). COMPASS-like core proteins exert the various biological functions on cancer progression, stem cell differentiation, and chromatin maintenance (24–30). However, the molecular mechanism of COMPASS-like core subunits targeting the specific chromatin regions to regulate gene expression in breast cancer remains poorly understood.

Histone readers are specific proteins that could interact with modified histone tails during gene transcriptional process, and several H3K4me3 readers have been identified and characterized, for instance, SGF29 is essential for linking the human SAGA complex to H3K4me3 (31–34). Bromodomain PHD Finger Transcription Factor (BPTF) associated protein of 18kDa (BAP18) encoded by *chromosome 17 open reading frame 49* (*C17orf49*) is also identified as an H3K4me3 reader (34). In the N-terminus of BAP18, there was a SANT (Swi3, Ada2, N-CoR, (TF) IIIB) domain, which usually exists in the chromatin-associated protein complex (35–38). We have previously identified BAP18 enhances androgen receptor action and promotes prostate cancer progression (39). However, as a histone H3K4me3 reader, the whole genomic occupation of BAP18 underlying the modulation of gene transcription needs to be further explored.

In this study, we provided evidence that BAP18 contributes to a poorer survival rate in breast cancer patients, especially in ERα-positive breast cancer patients. Genomic analysis has demonstrated that the majority of BAP18 binding sites are localized at promoter regions. BAP18-enrichment genes with estrogen treatment are involved in several tumor-related signaling pathways. Half estrogen response elements (EREs) are the significant enrichment sites found in estrogen-induced BAP18 binding sites. While the CCCTC binding factor (CTCF) and BORIS recognizing sites are the most obvious enrichment sites detected in BAP18 binding sites in the absence or presence of estrogen. BAP18 is recruited to the promoter regions of estrogen-induced genes, accompanied with the enrichment of H3K4me3 and H4ac. Moreover, BAP18 interplays with the endogenous ERα and co-activates ERα-mediated transactivation. Furthermore, BAP18 is required for the recruitment of COMPASS-like subunits to estrogen-response elements (EREs) in ERα target genes, thereby increasing H3K4me3 and histone acetylation levels. In addition, BAP18 promotes cell growth and associates with the sensitivity of ERα antagonist in ERα-positive breast cancer cells. Taken together, our data indicate the importance of a histone reader in breast cancer progression with potential future therapeutic implications.

## MATERIALS AND METHODS

### Cell lines and cell culture

All breast cancer cell lines were obtained from the cell bank of ATCC. T47D and BT474 were cultured in RMPI-1640 medium (Gibco). MCF7 was cultured in DMEM medium (Thermo Scientific). All cells were cultured with 10% fetal bovine serum (Gibco), 50 units/ml penicillin, and 50 units/ml streptomycin at 37°C and 5% CO_2._ 17β-estradiol (E2, Sigma-Aldrich) was dissolved in ethanol (Aladdin). All cells were cultured in phenol red-free DMEM or RMPI-1640 When it was needed to add E2 stimulus. Culture dishes were purchased from Guangzhou Jet Bio-Filtration Co., Ltd., China.

### Plasmids and antibodies

All plasmids about BAP18 were introduced in our previous work (39). ERα expression plasmid was cloned into pSG5 plasmid and 3 estrogen response elements were cloned made into pGL-ERE-adML reporter plasmid to perform luciferase reporter assay.

The antibodies were used in our study as follow: anti-BAP18 (Bethyl #A304-207A-1), anti-ERα (Cell signaling #D8H8), anti-MYC (Thermo Fisher #9E10), anti-CyclinD1 (Cell signaling #DCS6), anti-GAPDH (Kangchen #KC5G4), anti-WDR5 (Bethyl #A302-429A-2), anti-ASH2L (Bethyl #A300-107A-2), anti-DPY30 (Abcam #ab187690), anti-H3K4me3/H3ac/H3K9ac/H4ac/H4K16ac (Millipore), anti-Rabbit/Mouse (ABclonal), anti-IgG (Proteintech#10238-1-AP), anti-GFP (Sigma #G1544) and anti-FLAG (Proteintech#20543-1-AP).

### siRNA and lentivirus

siRNA and lentivirus of BAP18 were introduced in our previous work (39). siRNA duplexes against WDR5, ASH2L and DPY30 were purchased from Sigma. All sequences were listed in Supplementary Table S1.

### ChIP-sequencing (ChIP-seq) and data analysis

For BAP18 ChIP-seq, MCF7 cells were contained in a 9 ml DMEM medium fixed with 243 ul 37% paraformaldehyde (Electron Microscopy Sciences, 15714) and crosslinked for 15 min at room temperature. Then the crosslinks were stopped by 2.5 M glycine to a final concentration of 0.125 M. Cells were rinsed with icily PBS and performed assays with the help of Wuhan Seqhealth Tech Co. Ltd.

The primary analysis of ChIP-seq: The threshold of the number of the valid peak was selected within a false discovery rate (FDR) of 0.01. We used the default parameters from HOMER to distinguish genomic distribution, while promoter peaks were defined as those with peak center falling between 100bp downstream and 5000 bp upstream of transcript start sites (TSS). Exon, intron, and transcript terminate site (TTS) regions were exported from the National Center for Biological Information (NCBI), and the other regions were suggested as intergenic regions. To define meaningful BAP18 binding sites, we defined the fold enrichment of a peak was larger than 2 in IP versus input, and the fold change (FC) of tag density was considered at least *P* < 10e−2. Enrichment peak was then generated by R software (https://www.r-project.org) and measuring the meaning of these peaks was used Student *t*-test.

Other ChIP-seq data were downloaded from GEO datasets (https://www.ncbi.nlm.nih.gov/gds). ERα ChIP-seq data was from GSE45822; H3K27Ac data was from GSE62229; H3K4me1, H3K4me3, H3K9me3, H3K9ac and H3K27me3 data were from GSE23701; H3K4me2 data was from GSE24166; H3K36me3 data was from GSE39418.

### Luciferase reporter assays

HEK-293, MCF7, and T47D cells were co-transfected with BAP18 or its truncated mutants (200 ng), ERα (20 ng), ERE-tk-Luc (200 ng) and a plasmid of control Renilla luciferase (pRL) (5 ng) for series of luciferase assays. Cells were cultured into medium containing 5% charcoal-stripped fetal bovine serum (CS-FBS) with or without E2 stimulation after co-transfection 4 h. After an additional one day, cells were collected for dual-luciferase reporter assay (Promega).

### Western blotting and co-immunoprecipitation (co-IP) analysis

Western blotting assays were performed by the standard process introduced in our previous study (40). Immunoprecipitation analysis was started with whole-cell lysis purified with anti-IgG for 2 h before interaction. Protein G beads purchased from GE healthcare, concentrated into sepharose, were used for antibody-protein interaction rotating for overnight. Western blotting experiments were performed three times rinsing and lysis boiling.

### RNA and quantitative real-time PCR (qPCR)

Cells were prepared for RNA extraction after transfection negative control and siRNA against BAP18. After transfection 4–6 h, the final concentration of 100*n*M estrogen and equal EtOH were added to the culture medium for 16–18 h. Total RNA was extracted with RNA Trizol (TAKARA) and cDNAs were reversed by the PimeScript RT-PCR kit (TAKARA). Real-time qPCR assays were performed using the SYBR premeraseTaq kit (TAKARA) on LightCycler96 (Roche). All primers for qPCR were described in Supplementary Table S2, and statistics were performed by PRISM Graphpad 8. Every experiment was represented from at least three independent experiments and Student's *t*-tests were used.

### Chromatin immunoprecipitation (ChIP) and ChIP re-IP

Using standard protocols from Nature Protocols (41), we performed ChIP and ChIP re-IP. T47D cells were transfected with FLAG-tagged plasmids to overexpress BAP18 or siRNA to knockdown BAP18. Two kinds of cells were cultured into phenol red-free RPMI1640 with 10% CS-FBS for 2 days after transfection. With 80% confluency, the cells were treated with 100 nM E2 or equal EtOH for 12 h stimulation. After experiments, DNAs were used as templates for qPCR, and the primers were listed in Supplementary Table S3.

### Cell growth, colony formation and flow cytometry

All breast cancer cells were incubated for given periods and then harvest with trypan blue to count with a hematocytometer. Single-cell suspensions were performed with 100 cells per 35 mm dish and cell growth lines analyses for 1000 cells per column. Flow cytometry analyses were performed referring to our previous work (39).

### Drug resistance experiments

T47D cells were transfected with FLAG-BAP18 expression plasmids and treated with Geneticin-G418 (Gibco #1874962) to ensure all oeBAP18 cells stably overexpressed BAP18 more than normal T47D cells (Parental). Two kinds of T47D cells were counted for 2000 cells per 35mm dish and cultured for 21 days with different concentrations of 4-hydroxytamoxifen (Sigma #H7904), Fulvestrant (MCE #HY-13636) and Letrozole (MCE#CGS 20267). BT474 cells were treated with different dosages of Tamoxifen for 28 days and then stained with trypan blue to show survival cells.

### Animal experiments

The whole mouse experiments were performed at the Institutional Animal Care and Use Committee of China Medical University. T47D cells and BT474 cells were respectively stably expressed shCtrl and shBAP18 lentivirus. All cells were suspended in 50*u*l culture medium and 50 ul matrigel (BD biosciences) and injected the number of 5 × 10e6 into 4-week-old female *BALB/c* nude mice (Vital River Laboratory). Medication mice were treated with tamoxifen citrate (10 ug per tablet) every 3 days. Using the previous analysis measure, we monitored the mice every three days for about 4 weeks which time the mice were killed in keeping with the policy of humane treatment.

### Clinical samples and immunohistochemistry

All primary breast cancer tissues and adjacent tissues of patients were procured from the First Affiliated Hospital of China Medical University, all of which were got permission contents from patients already.

Breast tissue paraffin specimens were prepared from the First Hospital of China Medical University. The procedure of the experiment referred to our previous work (42) and the Ethics Committee of China Medical University approved this study.

## RESULTS

### The global genomic occupation of BAP18 upon estrogen treatment

**Figure 1.**
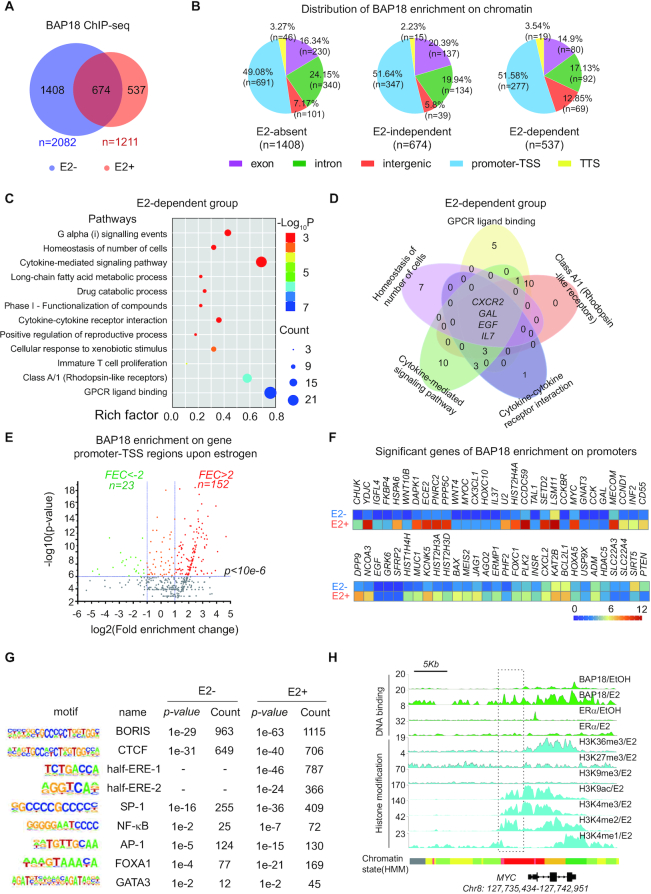
The global genomic occupation of BAP18 upon estrogen treatment. (**A**) MCF7 cells treated with or without estrogen (E2, 100 nM) were subjected to ChIP-seq with anti-BAP18 antibody, and overlapping between BAP18 ChIP-seq. Gene numbers of BAP18 binding in the presence or absence of estrogen were shown by the Venn diagram. (**B**) Genomic distribution of BAP18 binding sites on global chromatin among three groups: E2-absent, E2-independent, and E2-dependent. The percentage numbers represent the counts of genes in each region. (**C**) Bubble diagrams indicated related signaling pathways of all BAP18-enrichment-on-promoter genes among the E2-dependent group. The bubble color represented the *P*-value, and the bubble size represented the number of genes in the relevant pathway. Rich factor = gene in GO/gene in hit list × 100%. (**D**) Genes participated in GO pathways to merge with the Venn diagram, which showed the most important genes of BAP18-enrichment and their signaling among the E2-dependent group. (**E**) Volcano plot analysis screened out significant BAP18-binding genes on promoters-TSS upon estrogen treatment. Fold enrichment change (FEC) meant the fold enrichment of genes under estrogen treatment minus those under no treatment. Green plots were FEC←2 and red plots were FEC>2. (**F**) The heatmap showed the important BAP18-binding-promoter genes. The closer the color is to red, the higher of BAP18 peaks in the promoter region. (**G**) Motif analysis exhibited the potential transcription factors (TFs) which close to BAP18 recruitment on chromatin with or without E2 treatment (±100 bp from the center of ChIP-seq binding sites). Counts mean the number of promoters corresponding to each found motif on promoter-TSS regions. (**H**) Genomic browser snapshots, depicting BAP18 and ERαtwo proteins enrichment in the presence or absence of estrogen (green), and several histone modifications, including H3K36me3, H3K27me3, H3K9me3, H3K9ac, H3K4me3, H3K4me2 and H3K4me1 (Blue) with estrogen-induced on the *MYC* gene regions as shown. Boxed regions indicated considered BAP18-binding 5′ upstream regions. Genomic coordinates and read counts are indicated above.

Histone H3K4 tri-methylation (H3K4me3) on the promoter regions is usually supposed to be related to gene activation (34). It was previously reported that BAP18 was identified as a novel H3K4me3 reader with unknown function. We thus turned to perform Immunohistochemistry experiments in tissue microarray carrying the information of breast cancer progression to identify the relationship between BAP18 expression intensity and survival of breast cancer patients in 150 months. Results showed that patients with high expression of BAP18 had a poor prognosis compared with patients with low expression of BAP18 (Supplementary Figure S1A and S1D-E). Furthermore, measuring ERα status in these patients, we found that BAP18 contributed to poorer survival in ERα-positive breast cancer and had minimal impact on the survival of ERα-negative breast cancer patients (Supplementary Figure S1B, C). These observations prompted us that expression of BAP18 may be an important factor for determining patient survival and BAP18 probably was involved in the modulation of ERα-mediated transcriptional programs in breast cancer. ChIP-seq was further performed with antibodies against BAP18 to detect the occupation of BAP18 on global chromatin in response to E2 in MCF7 cells. As shown in Figure 1A, we defined all BAP18-enrichment genes into three parts: 1408 of E2-absent genes, 674 of E2-independent genes, and 537 of E2-dependent genes. 1211 BAP18 binding sites were strongly induced upon estrogen treatment (fold induction>2). According to the comparison of the NCBI genome sequence, more than half of BAP18 binding peaks were located in the promoter-TSS regions among three groups (Figure 1B). Pathways analysis (including GO and KEGG analysis) for BAP18-binding genes revealed the potential function of these genes. The results suggested that BAP18-enrichment genes among E2-dependent group mainly participate in homeostasis of the number of cells,cytokine-mediated signaling pathway, drug catabolic process, immature T cell proliferation and GPCR ligand binding (Figure 1C); while we merged signaling pathways according to rich factor and found that CXCR2, GAL, EGF and IL7 were probably involved in all five different pathways (Figure 1D). Furthermore, similar analyses were performed among the E2-absent gene column and E2-independent column. All gene oncology results indicated that BAP18 might participate in the modulation of the signaling pathways for tumor progression, cell cycle and gene transcription no matter estrogen presence or absence (Supplementary Figure S2A–D).

For further investigating and filtrating more significant genes, we used volcano plot analyses to screen out estrogen-induced genes, on which fold enrichment change significantly increased (FEC>2, *P*-value < 10e−4) (Figure 1E). A series of BAP18-enrichment genes upon estrogen treatment were screened as shown in Figure 1F, and we found that BAP18 could be recruited to the promoter regions of well recognized E2-induced genes, such as *MYC* and *CCND1*. Sequence analysis of BAP18 binding sites indicated that the half estrogen-response elements (EREs) as well as SP-1, NF-kB or AP-1 binding sites are the significant enrichment sites found in estrogen-induced BAP18 binding sites. Interestingly, the binding sequences for BORIS and the CCCTC binding factor (CTCF) are the most obvious enrichment sites detected in BAP18 binding sites in the presence or absence of estrogen (Figure 1G). The chromatin organizing factors, CTCF and BORIS, have been identified to be involved in numerous aspects of chromosome function, including chromatin insulation, enhancer blocking, transcriptional activation, DNA methylation-sensitive parental imprinting and DNA-loop formation between transcriptional control elements (43–46). Eventually, we turned to examine histone modification states on BAP18 enrichment sites of several ERα target genes, such as *MYC*, *CCND1, FOXC1* and *EGF*. The enrichment of BAP18 or ERα binding on the promoter regions was accompanied by the high level of histone H3K4me3 and H3K9ac, while low level of H3K9me3, H3K4me1, H3K36me3 and H3K27me3 in the presence of E2 (Figure 1H and Supplementary Figure S2E−G). These results suggest that BAP18 is probably recruited together with ERα to the promoter regions of estrogen-induced genes.

**Figure 2.**
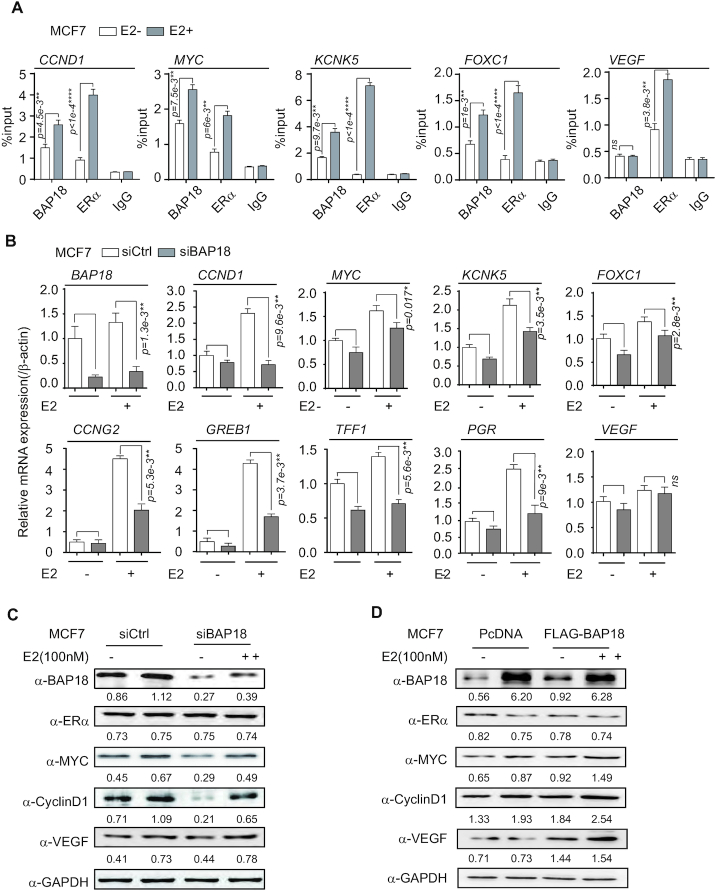
BAP18 is recruited to the promoter regions to activate the transcription of a series of genes. (**A**) The recruitment of BAP18 or ERα was examined by ChIP assay in MCF7 cells treated with or without estrogen (E2, 100*n*M). Quantitative RT-PCR (qPCR) analyses were performed with primers designed for recognizing 5′ upstream sequences of selected estrogen-induced genes as indicated. (**B**) qPCR analysis was examined for demonstrating the mRNA expression of estrogen-induced genes in MCF7 cells transfected with control siRNA (siCtrl) or siRNAs specific against BAP18 (siBAP18). Statistical significance was determined using Student *t*-tests. Error bars represent mean±SD. *P<0.05, **P< 0.01, ***P< 1e−3, ****P< 1e−4 and *ns* stands for no significance. (**C**) The effect of BAP18 depletion on the expression of estrogen-induced genes. MCF7 cells were transfected with control siRNA (siCtrl) or siRNA specific against BAP18 (siBAP18)in the presence or absence of estrogen (E2, 100 nM). (**D**) The effect of over-expressed BAP18 on the expression of estrogen-induced genes. MCF7 cells were transfected with PcDNA3.1 or FLAG-tagged BAP18 over-expression plasmids (FLAG-BAP18) in the presence or absence of estrogen (E2, 100 nM). The numbers below every grayscale image represented the ratio of protein expression/GAPDH expression in western blotting assays. All figures about qPCR and western blotting represented the results of three independent experiments.

ChIP assays were further performed to assess the binding of BAP18 on the upstream of transcription start site (TSS) of a series of genes in ERα-positive breast cancer cells. Consistent with the BAP18 enrichment regions screened from ChIP-seq analysis, we additionally provided the evidence to demonstrate that BAP18 was recruited to the *cis*-regulatory elements of several estrogen-induced genes, such as *CCND1*, *MYC*, *KCNK5*, *FOXC1*, except *VEGF* in MCF7 cells and T47D cells (Figure 2A and Supplementary Figure S3B). Thus, we turned to ask whether BAP18 might participate in estrogen-induced gene transcription. Quantitative RT-PCR (qPCR) was performed to examine the modulation of BAP18 on mRNA expression of estrogen-induced genes in ERα-positive breast cancer cells. We found BAP18 depletion decreased the mRNA expression of a series of genes, including *CCND1, MYC, KCNK5, FOXC1, CCNG2, GREB1, TFF1* and *PGR* in MCF7 and T47D cells in the presence of estrogen (Figure 2B and Supplementary Figure S3C). Furthermore, western blotting results showed that BAP18 depletion decreased MYC and CyclinD1 protein expression in MCF7 cells (Figure 2C). Meanwhile, the similar experiments were performed in MCF7 cells transfected with expression plasmids of BAP18, we found that MYC and CyclinD1 proteins were extremely increased by ectopic expression of BAP18 (Figure 2D). Taken together, the global occupancy of BAP18 analysis suggests that BAP18 can be recruited to the *cis*-regulatory regions of a series of estrogen-induced genes to maintain the activate transcription states.

### BAP18 interacts with ERα to enhance ERα-mediated transactivation

**Figure 3.**
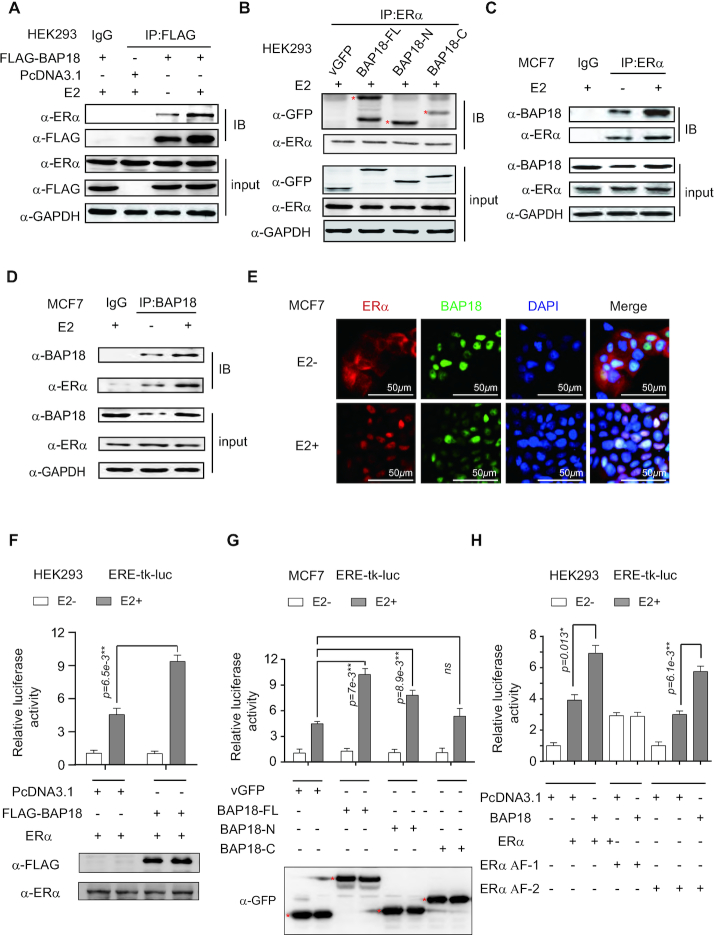
BAP18 associates with ERα to promote ERα-mediated transactivation. (**A**) HEK293 cells were co-transfected with PcDNA3.1/FLAG-BAP18 and ERα plasmids with or without estrogen (E2, 100 nM). Whole-cell extracts were immunoprecipitated with the anti-FLAG antibody or IgG after transfection for two days. Input means 5% of the whole extract for each column. (**B**) HEK293 cells were co-transfected with ERα and truncated mutant of BAP18 expression plasmids. All BAP18 expression plasmids carried GFP-tagged and vGFP plasmids were used for the control group. The location of the star indicates where the protein is expressed. BAP18-FL meant BAP18 full-length expression plasmid. BAP18-N, BAP18-C represented N-terminus of BAP18 expression plasmid and C-terminus of BAP18 expression plasmid. (**C**, **D**) Co-IP experiments showing the interaction between endogenous BAP18 and ERα in MCF7 cells. (**E**) The distribution of BAP18 and ERα with or without E2 by immunofluorescence staining in MCF7 cells. The cells were stained with anti-DAPI (Blue), anti-BAP18 (Green), and anti-ERα (Red). Scale bars were 50*u*m. (**F**) BAP18 enhances ERα-induced transactivation in HEK293 cells. The cells were co-transfected with ERα expression plasmid together with FLAG-tagged BAP18 expression plasmid (FLAG-BAP18) or PcDNA3.1 plasmid with (white histograms) or without (gray histograms) E2 treatment (100*n*M). The expression levels of FLAG-BAP18 or ERα were detected with anti-FLAG and anti-ERα by western blotting. (**G**) MCF7 cells were co-transfected with full-length BAP18 or its truncated mutants as indicated. The expression levels of BAP18 and its truncated mutants were examined with anti-GFP by western blotting. (**H**) BAP18 increases ERα or ERα AF-2 mediated transcriptional activity. The expression plasmids of ERα full length or truncated mutants carrying ERα AF-1 or ERα AF-2 were transfected into HEK293 cells with or without BAP18 expression plasmid as indicated. Relative luciferase activity as shown above is the mean value at least three times. Student *t*-tests were used and error bars represent mean ± SD. *P< 0.05, **P< 0.01 *and* ns stands for no significance.

Having demonstrated that BAP18 is recruited to the *cis*-regulatory elements of several ERα-regulated genes, and participates in the transcription of these genes, these results suggest that BAP18 might be involved in the modulation of ERα signaling pathway. We thus turned to detect the association between BAP18 and ERα. ERα and FLAG-tagged BAP18 expression plasmids were co-transfected into HEK-293 cells for co-immunoprecipitation (co-IP) experiments. The results showed that ERα was precipitated with FLAG-tagged BAP18 and the interaction between ERα and BAP18 was stronger in the presence of estrogen (E2) (Figure 3A). Additionally, different GFP-tagged truncated mutants of BAP18 were co-transfected with ERα into HEK-293 cells. BAP18-ERα interaction was mainly observed on BAP18 full length (BAP18-FL) or N-terminus, but slightly on BAP18 C-terminus, suggesting N-terminus of BAP18 comprising a SANT-domain play a dominating role in association with ERα (Figure 3B). Western blotting was further performed to examine the expression of BAP18 and ERα in different breast cancer cell lines. The results showed that BAP18 is expressed in the putative breast cancer cell lines as indicated (Supplementary Figure S3A). The endogenous-cellular co-immunoprecipitation (co-IP) experiments were performed to detect the endogenous interaction between BAP18 and ERα in MCF7 cells. In agreement with the above results, we observed that BAP18 is associated with ERα in MCF7 cells (Figure 3C, D). Moreover, immunofluorescence (IF) experiments were conducted to access the subcellular distribution of BAP18 and ERα in MCF7 cells. Our results showed that BAP18 was distributed in the nucleus with or without the treatment of E2, and BAP18 was co-located with ERα in the nucleus with the treatment of E2 in MCF7 cells (Figure 3E). Collectively, these data suggest that BAP18 interplays with ERα in cultured cells.

For exploring the function of BAP18 on ERα signaling pathway, luciferase assays were performed in cells. As shown in Figure 3F and G, the results demonstrated that BAP18-FL and its N-terminus mutant significantly up-regulated ERα-mediated transactivation in the presence of E2, but BAP18-C had no visible effect on ERα-mediated transactivation. The results suggest that BAP18 co-activates ERα action in a ligand-dependent manner and its N-terminus is probably required for its coactivator function on ERα action. To further examine which activation function domain of ERα could be regulated by BAP18, we performed luciferase assay to detect the effects of BAP18 on transcriptional activity induced by truncated ERα harboring the ligand-independent AF-1 domain (ERα AF-1) or ligand-dependent AF-2 domain (ERα AF-2). The results demonstrated that BAP18 up-regulated ERα AF-2-mediated transactivation in the presence of E2, while ERα AF-1 activation was not regulated (Figure 3H). Above all, our results suggest that BAP18 participates in the enhancement of ERα-mediated transcriptional activity.

### BAP18 facilitates the recruitment of core subunits of COMPASS-like complex to the promoter regions of ERα target genes

**Figure 4.**
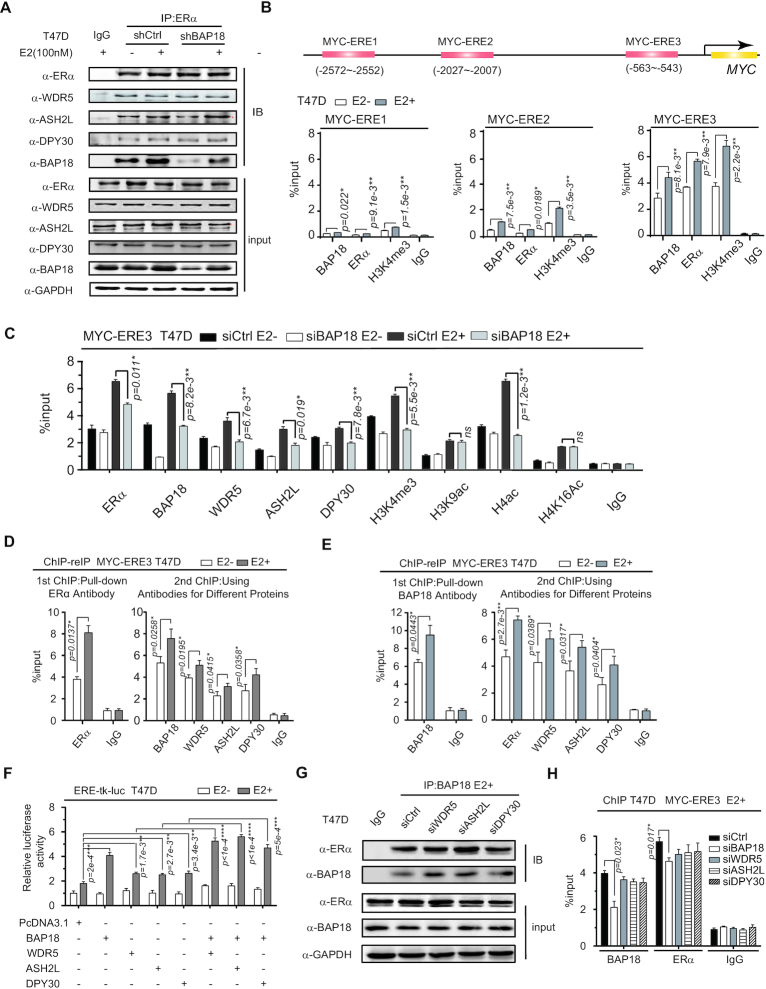
BAP18 facilitates the recruitment of COMPASS-like core proteins to the *cis*-regulatory elements of ERα target genes. (**A**) The Co-IP experiment was performed with anti-ERα and IgG in T47D cells carrying control shRNA or shBAP18 as indicated. Precipitated proteins were determined by western blotting with antibodies against COMPASS-like core proteins orBAP18/ERα as indicated. The location of the star indicates where the protein is expressed. (**B**) ChIP assays exhibited BAP18/ERα enrichments and modification of H3K4me3 at three independent EREs on upstream of the TSS region of the *MYC* gene. The location of EREs was indicated in the diagram above (red rectangle). (**C**) ChIP assays were performed with the antibodies as indicated by demonstrating the recruitment of proteins and the influence of BAP18 on protein recruitment and the histone modification levels as indicated on *MYC*-ERE3. T47D cells were transfected with control siRNA (siCtrl) or siRNA against BAP18 (siBAP18) with or without estrogen treatment (100 nM). (**D, E**) The recruitment of ERα (D) or BAP18 (E) together with each component of COMPASS-like core proteins on *MYC*-ERE3 by ChIP re-IP assays in T47D cells. (**F**) The additive effect of BAP18 on the co-activation function of COMPASS-like core proteins on the ERα-mediated transactivation. T47D cells were co-transfected with ERα together with BAP18, WDR5, ASH2L or DPY30 expression plasmids as indicated for luciferase analysis. FLAG-tagged PcDNA3 plasmids were used for the control group. (**G**) The depletion of each component of COMPASS-like core proteins does not affect the interaction between BAP18 and ERα by co-IP experiments in T47D cells. (**H**) The effect of COMPASS-like core proteins on the recruitment of BAP18 or ERα to the MYC-ERE region using ChIP assays. All data of ChIP assays, ChIP re-IP assays, and luciferase assays were representative of three independent experiments results. Student *t*-tests were used and error bars represent mean±SD. *P< 0.05, **P<0.01, ***P< 1e−3, ****P< 1e−4, *and ns* stands for no significance.

It has been previously reported that BAP18 as a histone H3K4me3 reader usually binds to tri-methylated histone tails on gene promoters. STRING protein network analysis suggests that BAP18 might associate with ERα and the subunits of COMPASS-like complex (COMPASS-like core proteins), including WDR5, ASH2L, and DPY30 (Supplementary Figure S4A). To determine the association between ERα and COMPASS-like core proteins together with BAP18, co-IP assays were performed in T47D cells. The results demonstrated that ERα associated with subunits of COMPASS-like core proteins together with BAP18. Also, the interaction between ERα and the subunits had no significant changes when BAP18 is depleted (Figure 4A). Meanwhile, the influence of depletion of BAP18 or COMPASS-like core proteins on the expression of other factors can be ignored as indicated (Supplementary Figure S4B, C).

To further analyze the molecular mechanism underlying the modulation function of BAP18 on the transcription of the estrogen-induced gene, such as *MYC*, we predicted 3 EREs upstream of transcription start site (TSS) of *MYC* by EnhancerDB homepage. ChIP assay results demonstrated that the modification level of H3K4me3 was the highest on ERE3, accompanied by the amount of BAP18 and ERα recruitment (Figure 4B). We thus turned to examine whether BAP18 or ERα is recruited to MYC-ERE3 together with COMPASS-like core proteins. ChIP assays were performed in T47D cells in estrogen presence or absence as indicated. The results demonstrated that BAP18 or ERα was recruited to the promoter region of *MYC*-ERE3 upon estrogen induction. Meanwhile, BAP18 depletion could respectively reduce the recruitment of ERα or the subunits of COMPASS-like complex to the same region. Also, BAP18 depletion decreased the levels of H3K4me3 and H4ac nearby the region of *MYC-*ERE3 and *TFF1* promoter in the presence of E2, while no significant effects of BAP18 on the levels of H3K9ac and H4K16ac were observed (Figure 4C and Supplementary Figure S4E). To confirm these results, ChIP assays were further performed in cells with the ectopic expression of BAP18. The results showed that overexpression of BAP18 facilitated the recruitment of ERα or COMPASS-like core proteins, thereby enhancing the levels of H3K4me3 and H4ac on the *MYC*-ERE3 region (Supplementary Figure S4D). ChIP re-IP assays were further performed to explore whether ERα and BAP18 could be recruited together with the subunit of the COMPASS-like complex to the promoter region of the ERα target gene. The results showed that ERα and BAP18 were recruited to *MYC*-ERE region together with COMPASS-like core proteins with estrogen presence (Figure 4D and E). The results indicated that BAP18 was able to facilitate ERα or the subunits recruitment to the promoter region of ERα target genes, thereby altering the levels of H3K4me3 to enhance gene transcription.

Luciferase assays results showed that BAP18 or the subunits of COMPASS-like core proteins respectively enhanced ERα-mediated transcription, and BAP18/WDR5, BAP18/ASH2L or BAP18/DPY30 could additionally co-activate ERα action in T47D cells (Figure 4F). Additionally, co-IP assays and ChIP assays suggested that the association between BAP18 and ERα, as well as the recruitment of BAP18 or ERα to *MYC-*ERE region was not able to be influenced by the depletion of each subunit of COMPASS-like core proteins (Figure 4G and H). Taken together, our results suggest that BAP18 associates with ERα and COMPASS-like core proteins to up-regulate ERα-mediated genes transcription in ERα-positive breast cancer cells.

### BAP18 promotes the cell growth in ERα-positive breast cancer cells

**Figure 5.**
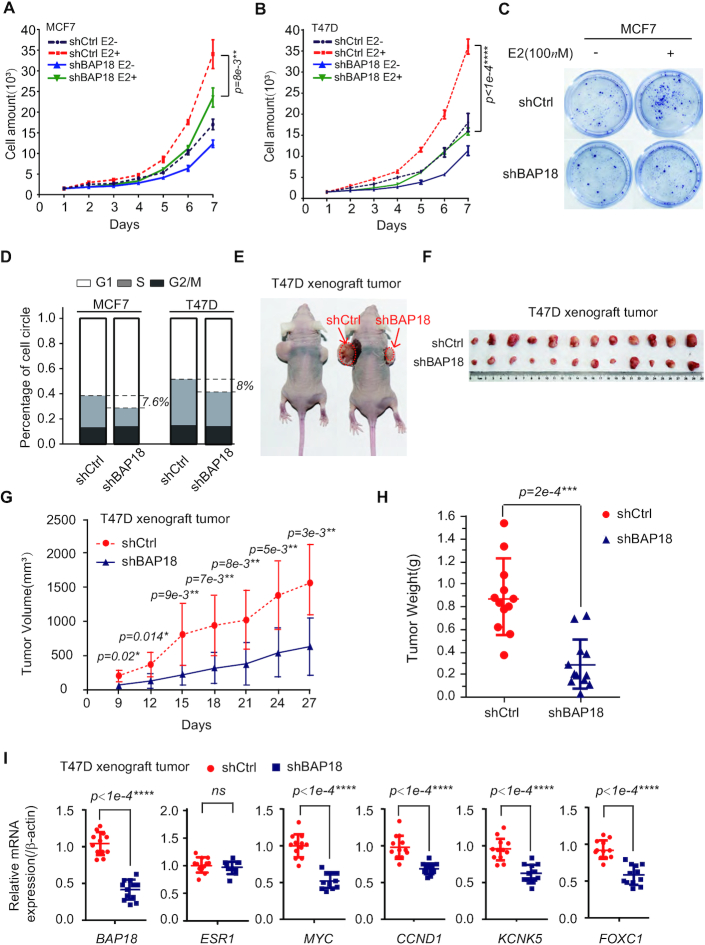
BAP18 promotes the cell growth in ERα−positive breast cancer cells. (**A**, **B**) The effect of BAP18 depletion on cell growth in MCF7 cells (A) or T47D (B) cells in the presence or absence of estrogen (E2, 100*n*M). Total cell amounts were enumerated every day, and error bars were represented as mean±SD of three independent experiments. (**C**) Representative photographs of clone formation assays with MCF7 cells under estrogen stimulation or not were shown. (**D**) The influence of BAP18 depletion on the cell cycle process in MCF7 cells or T47D cells by flow cytometry assays. The data were representative of three independent experiments. (**E**, **F**) Representative photographs showed the xenograft tumor in female *BAL B/c* mice injected with shCtrl (left/up) and shBAP18 (right/down) stable T47D cells in the normal feeding pattern. (**G**) The average tumor volume of shCtrl and shBAP18 were measured every three days beginning on the 9th day. (**H**) Tumor weights were measured between two groups of tumors 27 days later. (**I**) Quantitative RT-PCR analysis showed the mRNA level of BAP18, ERα and several ERα target genes in xenograft tumors. Student's *t*-tests were used in all data and error bars represent mean±SD. *P< 0.05, **P< 0.01, ***P< 1e−3, ****P< 1e−4 and *ns* stands for no significance.

Estrogen-ERα signaling pathway plays a key role in breast cancer processes, including tumorigenesis, tumor growth, carcinoma metastasis, and endocrine resistance. Having demonstrated that BAP18 acts as a co-activator of ERα to enhance estrogen-ERα mediated transactivation, we thus turn to examine the biological function of BAP18 in ERα-positive breast cancer cell lines, such as MCF7 cells and T47D cells. Growth curves analysis showed that knockdown of BAP18 inhibited cell proliferation in MCF7 cells and T47D cells (Figure 5A, B). Consistently, colony formation assays demonstrated that BAP18 depletion formed less of colonies than that in the control in the presence of estrogen (Figure 5C). Moreover, flow cytometry assays were performed as shown in Figure 5D, the results showed that depletion of BAP18 delayed the G1-S phase transition for about 7.6% or 8% in MCF7 or T47D cells. Collectively, the results suggest that BAP18 promotes cell growth/proliferation in the ERα-positive breast cancer cells.

To investigate the effect of BAP18 *in vivo*, the ectopic xenograft tumor model was established using T47D cells infected with lentivirus carrying shBAP18 or negative control lentivirus (shCtrl) separately. 4-week-old female *BAL B/c* nude mice (*n* = 12) were injected two kinds of infected cells at two oxters and the tumor volumes were measured every 3 days after injection. Compared with the control, our results suggested that BAP18 depletion resulted in depressed tumor growth before the control reached comparable sizes of tumors that had to be killed. Moreover, the tumor volumes from shBAP18-T47D cells demonstrated a slower growth rate than those from the control cells and the average tumor weight was lower than that from the control cells (Figure 5G, H). These results prompted us to performed quantitative RT-PCR to examine the changes in estrogen-induced genes. As shown in Figure 5I, knockdown of BAP18 decreased *MYC*, *CCND1*, *KCNK5*, and *FOXC1* mRNA expression in xenograft tumor tissue. Our results indicated that BAP18 could rapid tumor growth in mice and decreased estrogen-induced genes *in vivo*.

### BAP18 associates with the sensitivity of antiestrogen in ER-positive breast cancer cells

**Figure 6.**
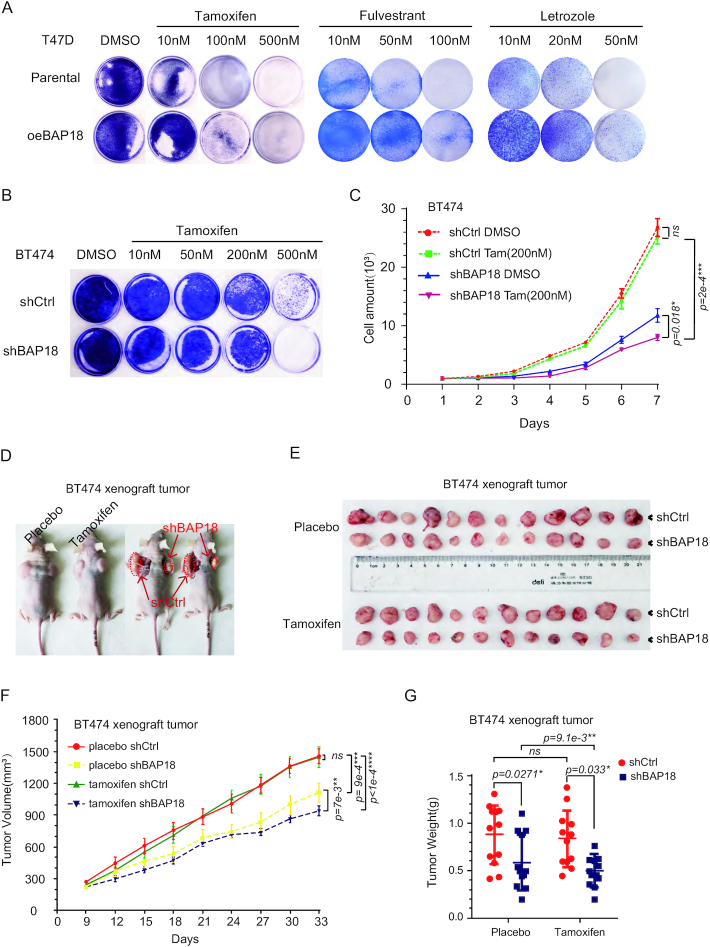
BAP18 enhances endocrine drugs sensitivity in ER-positive breast cancer cells. (**A**) The influence of BAP18 on endocrine drug sensitivity in T47D cells. T47D cells were pre-transfected with FLAG-tagged PcDNA3 plasmids (Parental) and BAP18 expression plasmids (oeBAP18). Cultured in different concentrations of three kinds of endocrine drugs for 14 days, cells in wells were stained by R250 and photographs represented one of three independent experiments. (**B**) The influence of knockdown of BAP18 on tamoxifen sensitivity in BT474 cells. Cells were infected with lentivirus against BAP18 (shBAP18) and cultured with a dose-dependent concentration of tamoxifen in a normal culture base. (**C**) Cell growth curves showed the effect of BAP18 depletion on tamoxifen treatment on cell growth in the BT474 cells. 200*n*M tamoxifen was used in 7 days’ culture. Student's *t*-tests were used in all data and error bars represent mean ± SD. *P< 0.05, **P< 0.01 and ***P< 1e−3. (**D**, **E**) Representative photographs for the xenograft tumors in female *BAL B/c* mice injected with shCtrl (left/up) and shBAP18 (right/down) stable BT474 cells, control cohort feed with placebo tablets and experimental cohort mice feed with tamoxifen citrate. (**F**) Tumor volume among the four groups showed the effect of BAP18 silencing on BT474 intrinsic Tamoxifen sensitivity. Mice were fed with equal of placebo and tamoxifen citrate in every three days. The drugs were used onthe9th day and the plot represents the volume of tumors. (**G**) Data showing the tumor weight of mice after 33 days. In all data, Student *t*-tests were used. Error bars represent mean ± SD. *P< 0.05, **P< 0.01, ***P< 1e−3, ****P< 1e−4 and ns stands for no significance.

Generally, applicable endocrine treatment for ERα-positive breast cancer has been usually adopted by using ERα inhibitors, including selective ERα modulators (such as Tamoxifen), aromatase inhibitors (such as Letrozole) and selective ERα degraders (such as Fulvestrant) in clinical therapy. We further evaluated the role of BAP18 in ERα-positive breast cancer cell survival in response to the treatment of ERα inhibitors. We first generated T47D cells with stable overexpression of BAP18 to observe how the parental T47D cells and derivative BAP18 expressing cells responded to Tamoxifen treatment. The results showed that the BAP18 expressing cells were at least two-fold more resistant in low concentration treatment, suggesting that BAP18 can reduce Tamoxifen sensitivity in T47D cells. We also treated the cells with Fulvestrant and Letrozole in appropriated concentration. Results showed that treatment of Fulvestrant and Letrozole led to the development of more resistant colonies among oeBAP18 groups, indicating that BAP18 weakened three kinds of endocrine drug sensitivity in T47D cells (Figure 6A and Supplementary Figure S5D). Also, drug treatment cell curves showed similar results in T47D cells with suitable drug concentrations (Supplementary Figure S5A−C). All these results indicated that BAP18 may be associated with the sensitivity of endocrine drugs.

Considering that Tamoxifen is the most common endocrine drug, we conducted Tamoxifen treatment assays in BT474 cells, which were recognized to have intrinsic Tamoxifen resistance. After establishing BAP18 knockdown in BT474 cells with shRNA, we treated the parental and shBAP18 cells into discrete concentration tamoxifen for 3 weeks. The results showed that the decrease of BAP18 led to fewer cell survival, especially in 500 nM tamoxifen treatment though BT474 could survive with tamoxifen slowly (Figure 6B). For further confirming the function of BAP18 in tamoxifen resistance progress, we treated BT474 cells with tamoxifen and DMSO under BAP18 depletion (shBAP18) or negative control (shCtrl) in short time experiments. Excluding the influence of BAP18 depletion, the results indicated that BAP18 depletion increased the sensitivity of tamoxifen in BT474 cells and also declined the growth of BT474 cells under tamoxifen treatment within two weeks especially in high concentration tamoxifen (Figure 6C). Taken together, our results indicate that BAP18 might be involved in influencing endocrine sensitivity in ERα-positive breast cancer.

According to the previous biological function of BAP18 on tamoxifen resistance in cultured cells, we next sought to explore whether BAP18 affected tamoxifen treatment *in vivo*. BT474 cells transfected with lentivirus shBAP18 or shCrtl were injected into 4-week-old *BAL B/c Nude* mice (*n* = 12). In the process of tumor growth, we fed each mouse with tamoxifen citrate or placebo pellets to ensure our tamoxifen treatment. Our results showed that knockdown of BAP18 could retard tumor growth no matter tamoxifen-treated (Figure 6F). Compared with placebo treatment, tamoxifen treatment only modestly delayed tumor growth; however, BAP18 depletion could magnify the tamoxifen effect to make more BT474 cells dead (Figure 6G). Similar to the data in vitro, all these results indicate that BAP18 is associated with the sensitivity of antiestrogen in ERα-positive breast cancer cells.

### BAP18 is highly expressed in clinical breast cancer samples

**Figure 7.**
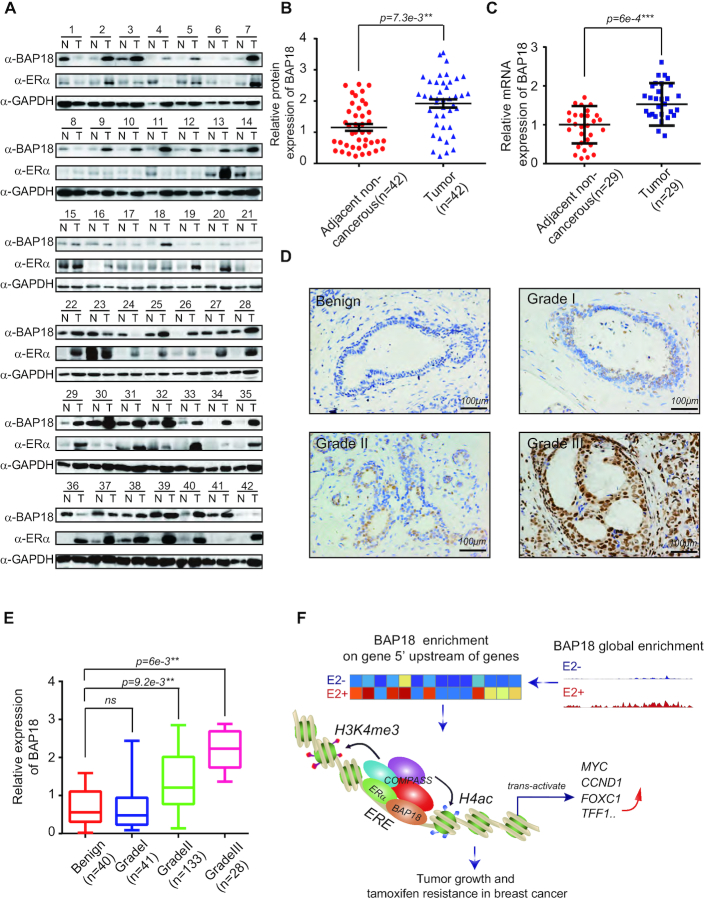
BAP18 is highly expressed in clinical breast cancer samples. (**A**) Expression of BAP18 and ERα in fresh human breast tissues. N represents adjacent noncancerous tissues and T for tumors. (**B**) Statistics for expression of BAP18 in adjacent noncancerous tissues and tumor tissues. The student *t*-test was used and **P< 0.01. (**C**) Statistics for expression of ERα in adjacent noncancerous tissues and tumor tissues. The student *t*-test was used and ***P<1e−3. (**D**) Representative tissue staining of BAP18 was shown. Magnification was 20× and the scaling bar was 100 μm. (**E**) the statistics of expression of BAP18 in different grades of benign tissue and breast cancer tissue. Student's *t*-tests were used and error bars represent mean ± SD. **P< 0.01 and ns stood for no significant. (**F**) The schematic representation of the function of BAP18 acting as a co-activation of ERα in breast cancer.

To investigate the expression of BAP18andERα in clinical breast cancer samples, we collected 42 pairs of freshly frozen breast cancer tissues and the adjacent noncancerous tissues, which were taken from the surgical operation. Especially in ERα-positive breast cancer, BAP18 had a higher expression level in cancer tissues than that in adjacent noncancerous tissues (Figure 7A-B). Also, the mRNA of BAP18 is high expressed in calculated 29 pairs of breast cancer individuals (Figure 7C). We also examined the expression of BAP18 and ERα in paraffin sections including 202 cases of breast cancer tissues and 40 cases of benign tissues using immunohistochemical (IHC) analysis. Compared with the low staining of BAP18 in benign tissues, BAP18 had an increased expression level in moderately differentiated breast cancer samples (Grade II) and poorly differentiated ones (Grade III) but had no significance in well-differentiated ones (Grade I) (Figure 7D-E). Furthermore, BAP18 expression exhibited a solid link with the pathological factors, indicating that BAP18 tended to bring superiority into ERα-positive breast cancer and deteriorated the process of cancer (Table 1). Our data suggest that BAP18 is highly expressed in breast cancer tissues, contributing to the promotion of cell growth and endocrine resistance in ERα-positive breast cancer.

**Table 1.** Expression of BAP18 in breast cancer samples with clinical characteristics.

Variables	Groups	Cases (*N* = 150)	BAP18 high expression (*N* = 96)	BAP18 low expression (*N* = 54)	*P*-value
**Univariate analysis**					
**Age**	≤55	94	67	27	
	>55	56	29	27	0.0162*
**T**	T1	33	17	16	
	T2+	117	79	38	0.0907
**N**	N0/N1	103	67	36	
	N2+	47	29	18	0.6921
**Histological stage**	I	28	9	19	
	II/III	122	87	35	<0.001****
**ERα status**	ERα+	119	84	35	
	ERα-	31	12	19	0.001***
**PR status**	PR+	68	41	27	
	PR-	82	55	27	0.3892
**HER2status**	HER2+	17	11	6	
	HER2−	133	85	48	0.9487
**Multivariate analysis**					
**Age**	≤55	94	67	27	
	>55	56	29	27	0.0873
**Histological stage**	I	28	9	19	
	II/III	122	87	35	<0.001****
**ERα status**	ERα+	119	84	35	
	ERα−	31	12	19	0.2455

T represents tumor size and N represents lymph Node.

In univariate analysis, *Chi-square* test were used, ***P< 0.001 and *P< 0.05; and in multivariate analysis, *Hosmer and Lemeshow* test were used, ***P< 0.001.

## DISCUSSION

BAP18 has been previously identified as a novel modifier of chromatin structure to be involved in the regulation of androgen receptor action using our *Drosophila* experimental system (39). It has been reported that BAP18 acts as an H3K4me3 reader with unknown function (34). Importantly, we recently found the significant high expression of BAP18 is positively correlated with the poor survival in ERα-positive breast cancer. However, the global enrichment of BAP18 on the chromatin upon estrogen stimulation, and the molecular mechanism underlying the function of BAP18 on ERα-positive breast cancer are largely unknown. Here, ChIP-seq analysis was performed to show that the half estrogen response elements (EREs) as well as SP-1, NF-kB or AP-1 binding sites are the significant enrichment sites found in estrogen-induced BAP18 binding sites. Moreover, we have demonstrated that BAP18 is a vital co-regulator for estrogen-induced gene activation. As an H3K4me3 reader, BAP18 is required for the recruitment of the core subunits of the COMPASS-like complex to the promoter regions of ERα target genes. BAP18 is involved in the promotion of tumor growth and proliferation and associates with the sensitivity of antiestrogen in ERα-positive breast cancer (Figure 7F).

Histone readers can specifically recognize histone markers, constituting a vital mechanism of maintaining the proper epigenetic landscape. Lysine methylation on histone is commonly associated with the regulation of gene transcription. H3K4me3 is a specific hallmark of active chromatin, and H3K4me3 reader usually acts as a component of the protein complex to be involved in epigenetic modulation of gene transcription to exert their vital biological functions. Several H3K4me readers have been identified to be essential for the regulation of gene transcription and tumor progression (47–54). Here, we focused on investigating the function of BAP18 characterized as a novel H3K4me3 reader in breast cancer. Consistent with the previous finding that BAP18 binds at H3K4me3 sites of histone, BAP18 was found in this study to be mainly enriched on a high-level of H3K4me3 sites of activated ERα target genes and BAP18 was mainly recruited to promoter regions of its enrichment genes with or without estrogen stimulation in MCF7 cells (Figures 4B and 1B). Thus, BAP18 appeared to be a critical determinant for promoter activation of genes.

From the genomic analysis of BAP18 occupation on the whole genome, ChIP-seq analysis has demonstrated that BAP18-enrichment genes participate in a series of important signaling pathways involved in the cancer process. Interestingly, BAP18 would be recruited to the promoter region of E2-induced genes, such as *MYC*, *CCND1*, and *FOXC1*, with ERα as well as other transcription factors, such as FOXA1, SP-1, AP-1 and GATA3. These findings suggest that BAP18 might be involved in regulating the transcription of putative E2-induced genes through other transcription factors, besides ERα. On the other hand, we found that the recognizing motifs of CCCTC binding factor (CTCF) and BORIS, which can link chromatin domains via long-distance interactions (55), are the most obvious enrichment sites detected in BAP18 binding sites. These findings suggest that in addition to the co-activation function of ERα action, BAP18 possibly participates in the loop formation of promoter-enhancer during gene transcription regulation process.

Histone methyltransferase complexes, mainly consisting of histone methyltransferases (HMTs) and histone interacting proteins, play crucial roles in chromatin remodeling, histone modification, cell differentiation, or tumorigenesis in mammalian cells. COMPASS-like core subcomplex is acceptable for H3K4 methyltransferase activity, which contains WDR5, RbBP5, DPY30, ASH2L, and other potential combinative proteins (56–60). Prediction analysis for the proteins which were potentially associated with BAP18 by STRING database, it is possible that BAP18 interacts with WDR5, ASH2L, and DPY30 in the presence of ESR1 (ERα). In T47D cells, we provided the evidence to show that BAP18 could interact with the COMPASS subcomplex with ERα, while had little effect on spatial interaction between COMPASS proteins and ERα. On the chromatin, BAP18 could influence ERα and COMPASS recruitment in specific DNA regions. These results indicate that BAP18, as an H3K4me3 reader, also acts as an adaptor of COMPASS complex, participating in ERα-mediated gene activation. Of interest, genome browser tracks of estrogen-induced genes have shown that BAP18 bound on gene proximal promoter region with activation hallmarks, such as H3K4me3 and H3K36me3 with estrogen treatment (Figure 1H). Regarding the regulation function of BAP18 as an H3K4me3 reader on ERα action, we speculate that there might be a first estrogen-induced activation status on the promoter regions of ERα target gene. Thereafter, BAP18 is recruited to recognize H3K4me3 at the active chromatin to contribute to continuously maintain ERα-induced gene activation. Meanwhile, BAP18 facilitates the recruitment of COMPASS-like core proteins for further altering histone modification levels at the promoter region of the ERα target gene. Also, the predicted binding proteins of BAP18 contained SETD1A, SETD1B, and NCOA1, which had been recognized as typical histone methyltransferase and acetyltransferase (Supplementary Figure S4A). These results suggest that BAP18 might be involved in complicated chromatin events to be essential for epigenetic modulation of ERα-mediated gene transcription in breast cancer.

The co-regulators of ERα are necessary for modulating ERα action to maintain homeostasis of ERα function. Abnormal regulation of ERα action leads to breast tumorigenesis or transformation of endocrine resistance. Some of ERα target genes, including *MYC*, *EGF* or *FOXC1*, play crucial roles in endocrine resistance (15,16,61–63). It has been reported that ERα-induced activation of enhancers/super-enhancers and transcriptional reprogramming has been recently reported to be crucial for the occurrence of endocrine resistance (64–66). Thus, identification of the novel co-regulator and clarification of the molecular mechanism for modulation of E2/ERα signaling in breast cancer is important to advocate a new therapeutic strategy for ERα-positive breast cancer and endocrine resistance. In our study, we provide evidence to show that BAP18 as a novel co-activator of ERα associates with the sensitivity of endocrine drugs. The potential molecular mechanism is speculated as followed: In this study, our data indicate that BAP18 acting as a co-activator of ERα enhances ERα-induced transactivation. BAP18 or ERα is recruited to promoter regions of putative ERα downstream regulatory genes (Figures 2 and 4). Moreover, BAP18-enrichment genes are mainly involved in cancer/endocrine resistance-related signaling pathway activation, such as G protein-coupled receptor (GPCR) ligand binding and cytokine-mediated signaling pathways in the presence of E2 (Figure 1C, D). Thus, BAP18 might alter the sensitivity of antiestrogen through its function on the regulation of ERα-mediated transcriptional activity; On the other hand, it would be the function of BAP18 on another important signaling pathway except for ERα. It has been reported that BT474 exhibited HER2 over-expression pattern, HER2 signaling pathway, and its associated pathways (including MAPK cascade, protein phosphorylation, cell surface receptor signaling pathway, tyrosine kinase signaling pathway, etc.) were compensatively activated with antiestrogen treatment (67,68). Using global ChIP-seq analysis, we found that BAP18-enrichment genes participate in a series of signaling pathways in E2-absent or E2-independent conditions. BAP18 would play its genome-wide roles in modulation of gene transcription via recruitment of other transcription factors, thereby participating in the regulation of some signaling pathways, including HER2 associated pathways, which are crucial for endocrine resistance (Supplementary Figure S2A–D).

Collectively, our data have demonstrated that BAP18 as an H3K4me3 reader participates in epigenetic modulation of ERα action, thereby promoting cell growth and proliferation in breast cancer-derived cell lines. Moreover, BAP18 is associated with the sensitivity of antiestrogen. These findings suggested that BAP18 may serve as a potential therapeutic target for ERα-positive breast cancer, and even for endocrine resistance. Thus, understanding the molecular mechanism of BAP18 in regulating oncogenic ERα pathway would be helpful to define the improved therapeutic strategies and develop small molecular reagents for endocrine-resistant breast cancer.

## Supplementary Material

gkaa787_Supplemental_Files
