# Supplementary material for: An H3K4me3 reader, BAP18 as an adaptor of COMPASS-like core subunits co-activates ERα action and associates with the sensitivity of antiestrogen in breast cancer
Source: Nucleic Acids Res. 2020 Sep 28;48(19):10768–84. doi: 10.1093/nar/gkaa787 (PMC7641737; doi:10.1093/nar/gkaa787)

Supplementary Figure 1

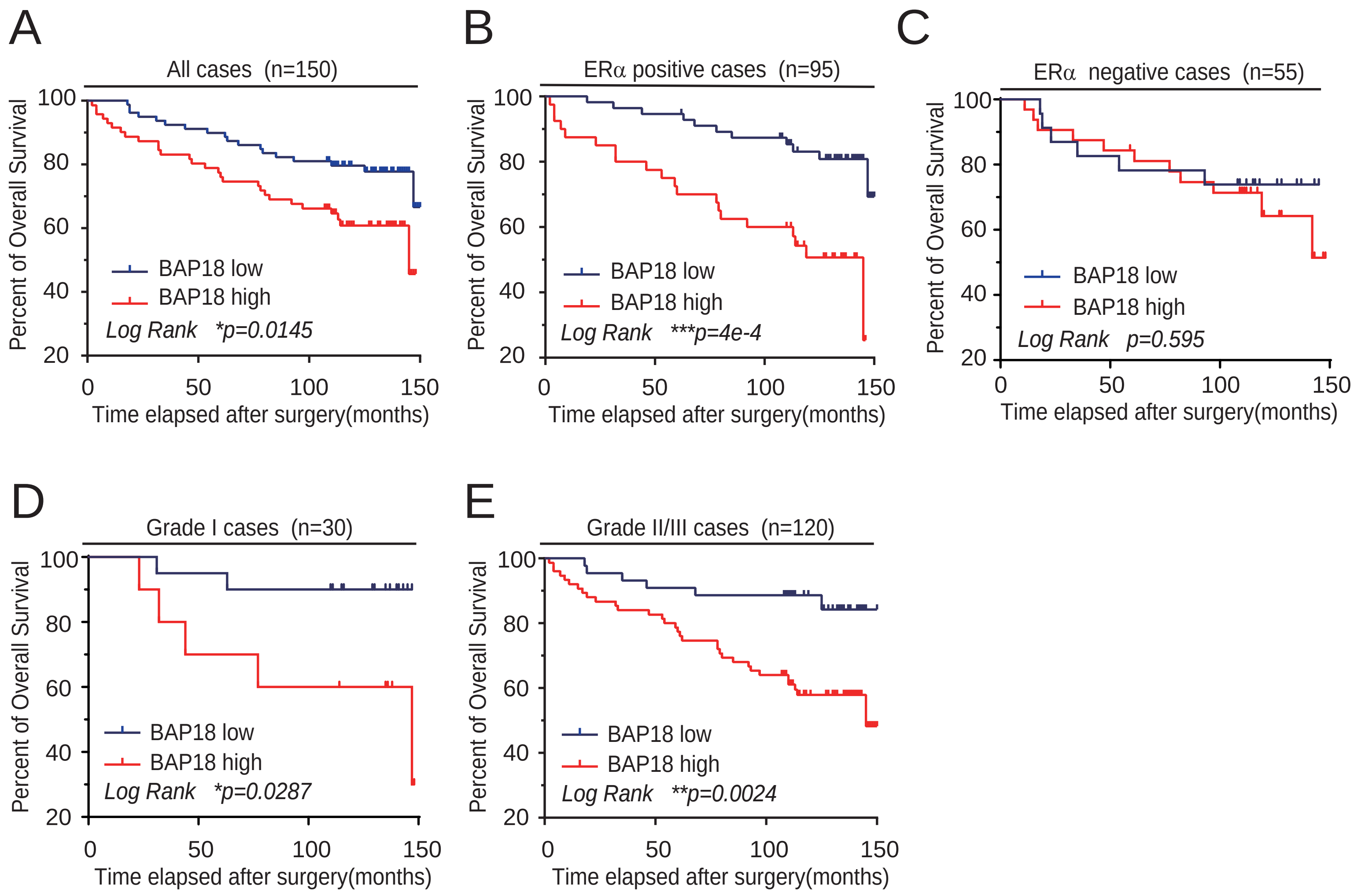

Supplementary Figure 2

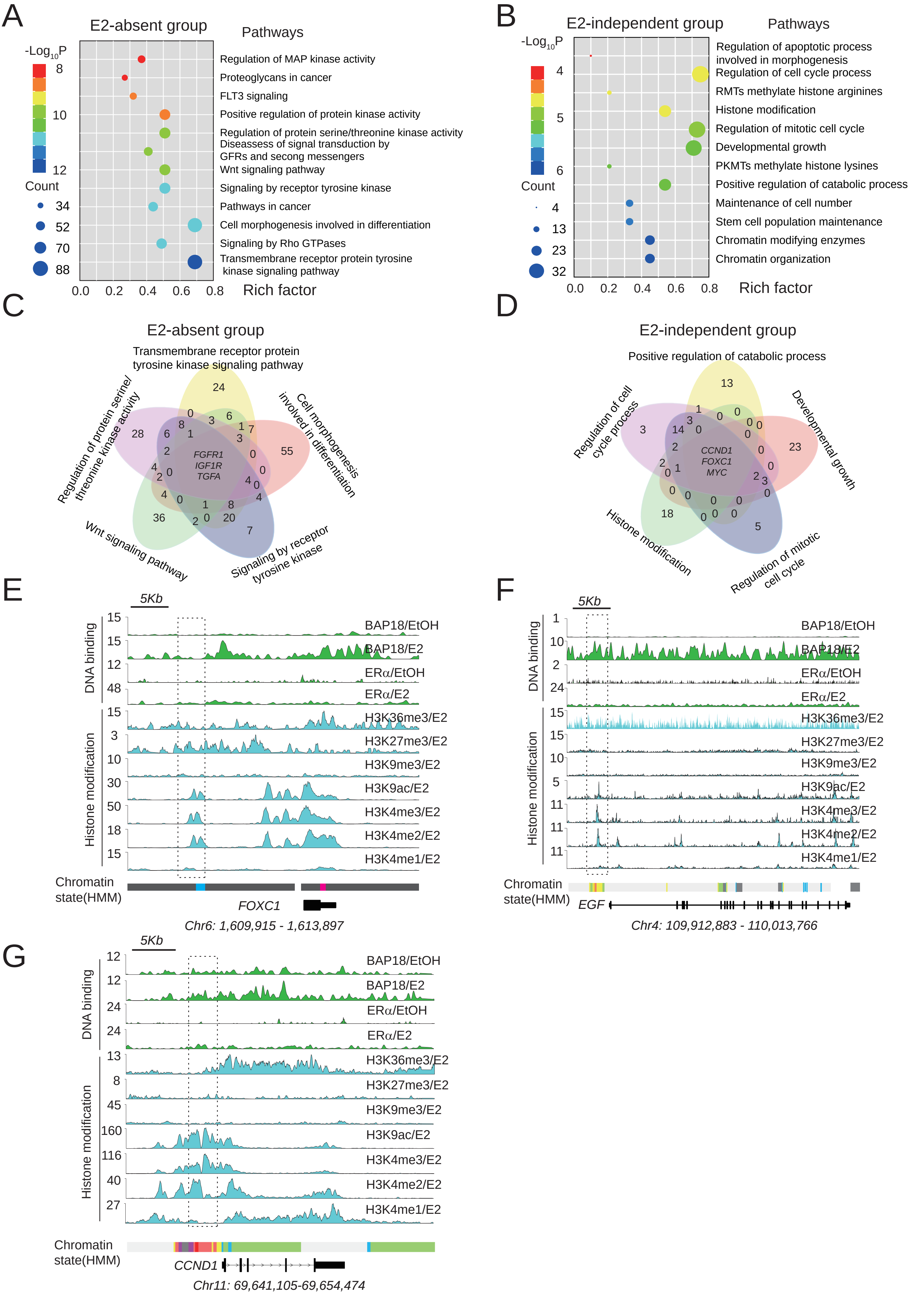

Supplementary Figure 3

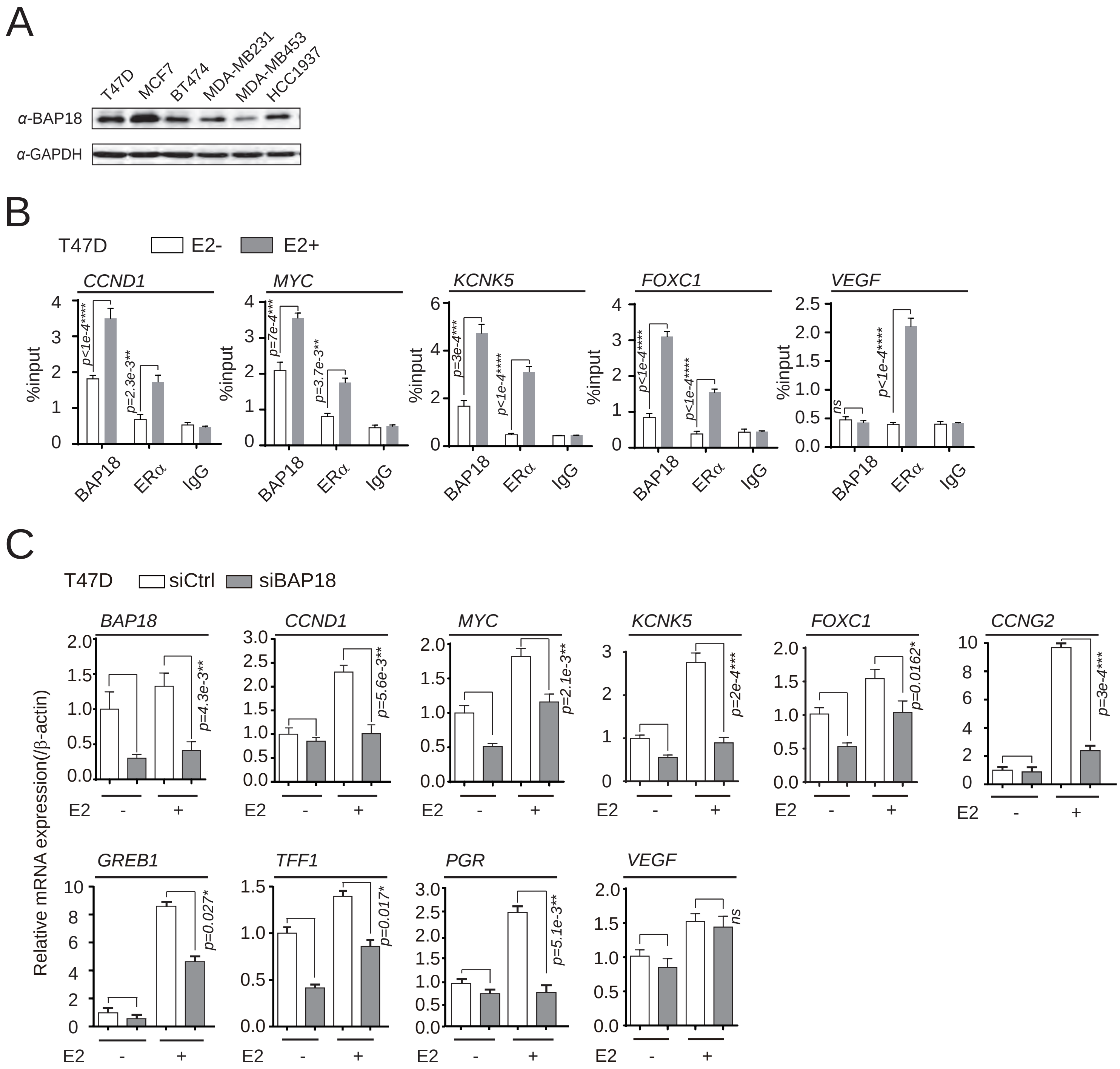

Supplementary Figure 4

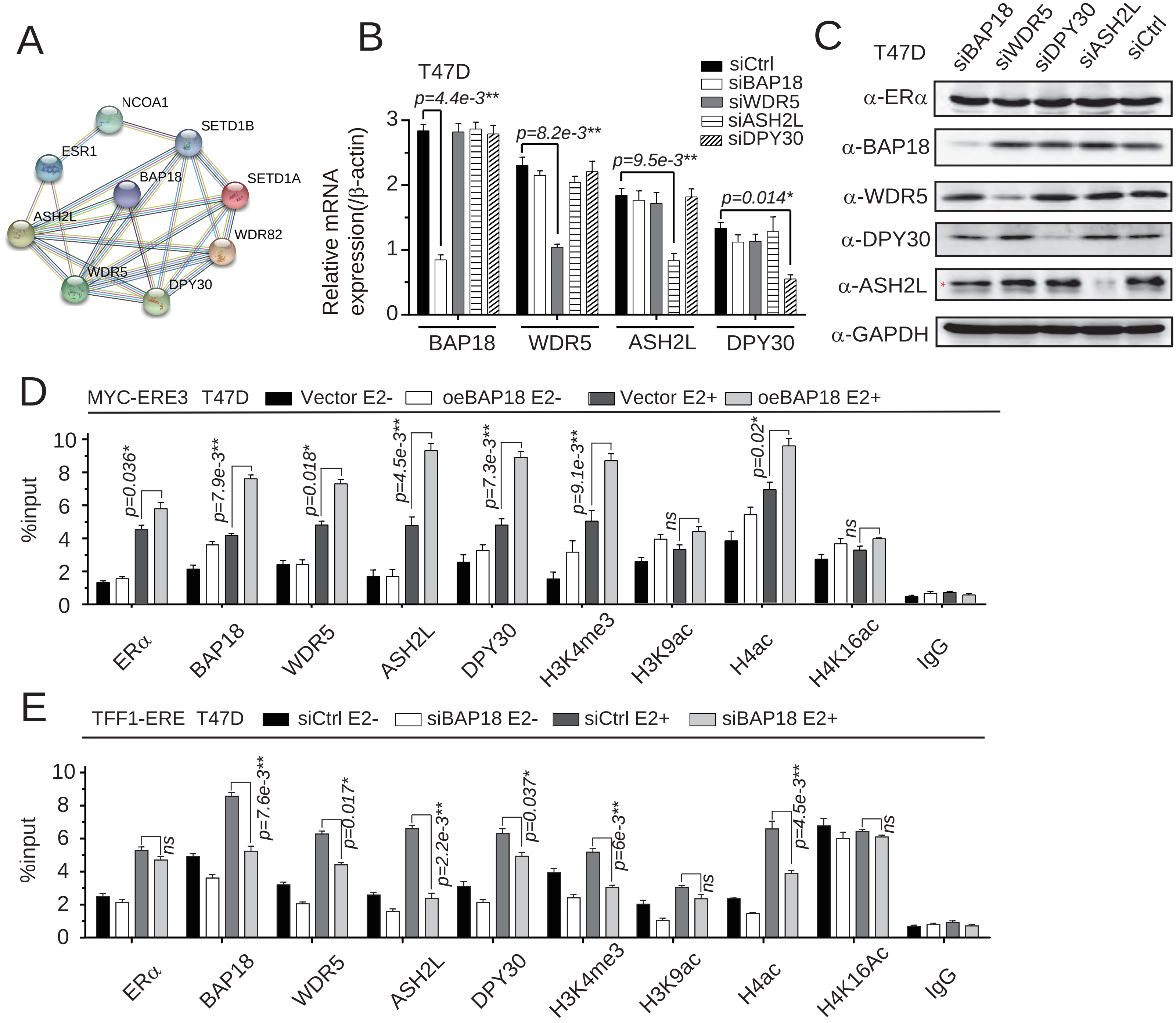

Supplementary Figure 5

A

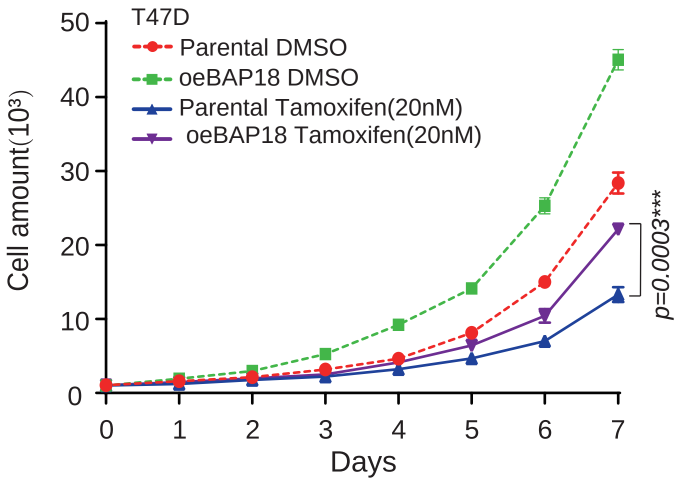

B

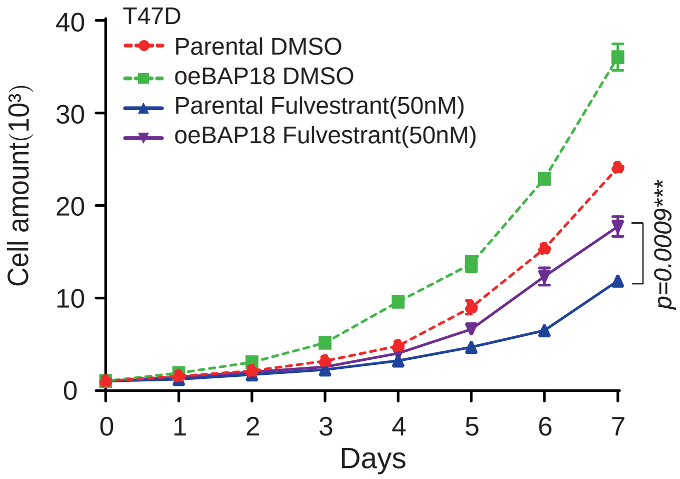

C

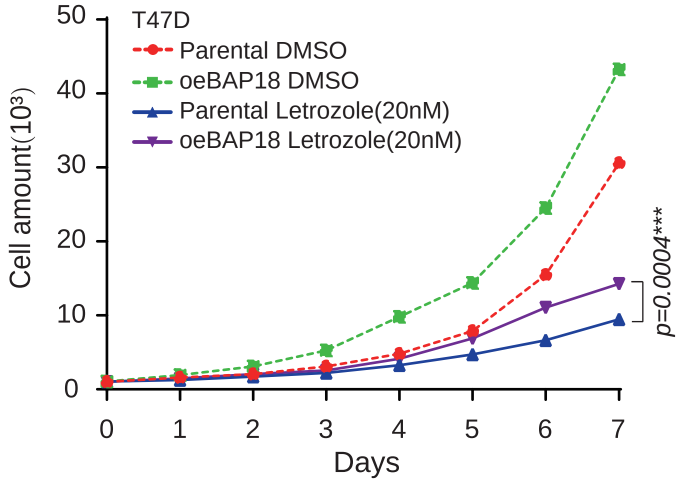

D

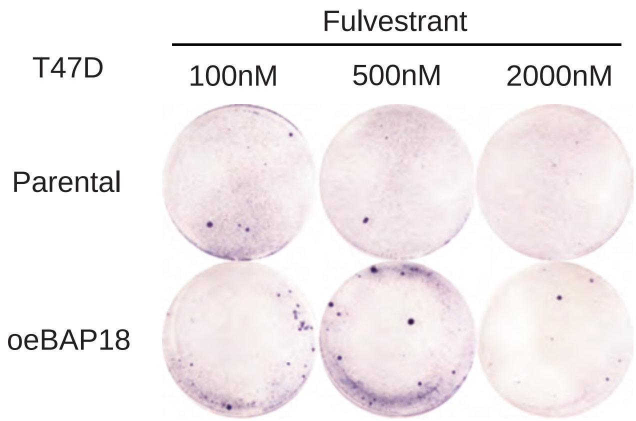

Supplement: gkaa787_Supplemental_Files [file gkaa787_supplemental_files.zip › Figure S1-5.pdf]
