# Supplementary material for: An H3K4me3 reader, BAP18 as an adaptor of COMPASS-like core subunits co-activates ERα action and associates with the sensitivity of antiestrogen in breast cancer
Source: Nucleic Acids Res. 2020 Sep 28;48(19):10768–84. doi: 10.1093/nar/gkaa787 (PMC7641737; doi:10.1093/nar/gkaa787)
Supplement: gkaa787_Supplemental_Files [file gkaa787_supplemental_files.zip › Supplementary figure legends-0820.docx]

**Supplementary data**

**Supplementary Figure Legends**

**Supplementary Figure 1. High expression of BAP18 is positively correlated with poor survival in breast cancer.**

(A-C) Overall survival analysis indicated the relationship between the BAP18 expression levels and patients' survival time among (A) all cases or (B) all ERα- positive cases or (C) all ERα-negative cases. (D-E) Overall survival showed the effect of BAP18 expression on patients who belong Grade I (D) and Grade II/III (E). *Log-rank* tests were used among these patients, **p<0.05, **p<0.01, ***p<1e-3,* and *p>0.05* meant no significant*.*

**Supplementary Figure 2. The global genomic occupation of BAP18 upon estrogen treatment.**

(A-B) Bubble diagrams indicated related signaling pathway of all BAP18-enrichment-on-promoter genes in E2-absent group (A) and E2-independent groups (B). (C-D) Genes among E2-absent group (C) and E2-independent group (D) participate in GO pathways to merge with Venn diagram, which showed the most important genes of BAP18-enrichment and their signaling. (E-G) Genomic browser snapshots, depicting BAP18 and ER binding and several histone modifications on *FOXC1* (E), *EGF* (F), and *CCND1* (G). Genomic coordinates and read counts are indicated above.

**Supplementary Figure 3. BAP18 is recruited to the cis-regulatory elements of a series of estrogen-induced genes.**

(A) Western blotting assay indicated the expression of BAP18 in six independent cell lines. (B) T47D cells were treated with or without estrogen (E2, 100*n*M), following quantitative RT-PCR (qPCR) analysis to examine the recruitment of BAP18 and ERα on selected estrogen-induced coding genes 5’ upstream sequencing as indicated. Student *t*-test were used for statistics and error bars represented mean±SD, ***p < 0.01, ***p < 1e-3, ****p<1e-4* and *ns* meant for no significant. (B) qPCR analysis demonstrating the mRNA expression of estrogen-induced genes in T47D cells transfected with control siRNA (siCtrl) or siRNAs specific against BAP18 (siBAP18). Student *t*-tests were used and error bars represent mean±SD. **p<0.05; **p<0.01, ***p<1e-3, ****p<1e-4* and *ns* for no significant*.* (C) ChIP assays were performed to show the enrichment of BAP18 and ERα on several BAP18-decreased-enrichment genes’ promoter regions. Student *t*-tests were used, **p < 0.05* and ***p < 0.01.*

**Supplementary Figure 4. BAP18 facilitates the recruitment of core subunits of COMPASS-like complex to the promoter regions of ERα target genes.**

(A) The interaction relationship between BAP18 and several COMPASS core proteins with ERα generating by STRING (https://string-db.org/). (B-C) Different siRNA against BAP18 or COMPASS proteins treated in T47D cells. (B) Statistics for q-PCR in mRNA expression and (C) for protein expression. Student *t*-tests were used and error bars represent mean±SD. **p<0.05*and ***p<0.01.* (D-E) Chromatin immunoprecipitation (ChIP) assays were performed with different antibodies indicating proteins recruitment or histone modification level. T47D cells were transfected with BAP18 overexpression plasmids on *MYC-ERE* (D) or siRNA against BAP18 on *TFF1-ERE* (E) with or without estrogen (10*n*M) and analyzed by q-PCR. Student*-t* tests were used, **p<0.05; **p<0.01 and ns* for no significant*.*

**Supplementary Figure 5. BAP18 alters the sensitivity of antiestrogen in T47D cells**

(A-C) cell curve assays showed the effect of BAP18 on three kinds of endocrine drugs with IC50 concentrations. Student *t*-test were used and ****p<1e-3*. (D) High level concentration of Fulvestrant were treated in parental and BAP18 over-expression (oeBAP18) T47D cells. Cells were treated about 14 days and stained with Coomassie brilliant blue.
