# Supplementary material for: An H3K4me3 reader, BAP18 as an adaptor of COMPASS-like core subunits co-activates ERα action and associates with the sensitivity of antiestrogen in breast cancer
Source: Nucleic Acids Res. 2020 Sep 28;48(19):10768–84. doi: 10.1093/nar/gkaa787 (PMC7641737; doi:10.1093/nar/gkaa787)
Supplement: gkaa787_Supplemental_Files [file gkaa787_supplemental_files.zip › TableS1-S3.docx]

**Supplementary table 1. siRNA used in this study**

| Name | Sense(F’) | Anti-sense(R’) |
| --- | --- | --- |
| BAP18 | GGGACGAUCUUAAUCACAUdTdT | AUGUGAUUAAGAUCGUCCCdTdT |
| DPY30 | CACUCUGAGUACGGUCUCAdTdT | UGAGACCGUACUCAGAGUGdTdT |
| WDR5 | CGAUGUAGCCUGGUCGUCAdTdT | UGACGACCAGGCUACAUCGdTdT |
| ASH2L | GAGAUGUAUUCUUGGUAAAdTdT | UUUACCAAGAAUACAUCUCdTdT |

dTdT: DNA bases within RNA oligos.

**Supplementary table 2. Primers used for quantitative RT-PCR**

| Name | Sense(F’) | Anti-sense(R’) |
| --- | --- | --- |
| BAP18 | TGGCATCTGGTGTCTTGTCA | TTGGCATCGGAGTCGTTCA |
| KCNK5 | GTCCTGTGTTCTTCCCGCTC | CCCTAGTCTCTTGGCACGTC |
| MYC | CGTCCTCGGATTCTCTGCTC | GCTGGTGCATTTTCGGTTGT |
| FOXC1 | AGTCAGCTTGCTTTGAGGCTA | AGGCATCACCGTGGTAAGAC |
| VEGF | GTCTTGACTCTACTCCACCCC | CTCGGTACTGACATCGCTCC |
| ESR1 | GACTGCACTTGCTCCCGT | CCACTTCGTAGCATTTGCGG |
| CCND1 | CTGATTGGACAGGCATGGGT | GTGCCTGGAAGTCAACGGTA |
| CCNG2 | GGTGAGGCTACAGTGATTCCA | CAAGGCACAGATGCCAAACC |
| GREB1 | ATGGAGGACCTGGAGCAGAT | ACAGTGCTACTCACAAGATCCC |
| TFF1 | TCCCCTGGTGCTTCTATCCT | GGACTAATCACCGTGCTGGG |
| HSP27 | AGACGTCCAGAGCAGAGTCA | ATGTAGCCATGCTCGTCCTG |
| PGR | AAATCTACAACCCGAGGCGG | CGGCTCCTTTATCTCCCGAC |
| WDR5 | GGCTGGCAAGTTCATCTG | TGAGACAATAAGGTTGGACTG |
| ASH2L | GTGGCTTGGAGACAGAAT | AGGTAGACAGGATGAGGTAT |
| DPY30 | TCACTCTGAGTACGGTCTC | GATAGGCACAACTGTCTGAT |

**Supplementary table 3. Primers of ChIP used for quantitative RT-PCR**

| Name | Sense(F’) | Anti-sense(R’) |
| --- | --- | --- |
| TFF1 | GGCCATCTCTCACTATGAATCACTTCTGC | GGCAGGCTCTGTTTGCTTAAAGAGCG |
| MYC | CTCCCGTCTAGCACCTTTGA | GATGTGTCTGCCTGTTCCAGA |
| KCNK5 | TGTTCTTCCCGCTCCTTACG | GCCCTCCAGCCTCTGAAAAC |
| FOXC1 | GACGAACCTTCTTCCCCACC | TCCTGTCCTGAACTCGGCTA |
| VEGF | CCTTTGCCTTGCTCTGTCAC | TGTTGTGCTGTTGTCCTCCA |
| MYC-ERE1  MYC-ERE2  MYC-ERE3 | CCCTGTGGAGAGCACTCATTT  TCACTTATTATTCACCAGCCCA  TTTTTGTGCATGACCGCATTTC | TACGGGGCAAAGAATCCCTG  GGTGATGGGTATTTGGTTTGGC  ACCGGACTTCCTAAAAGGGG |
